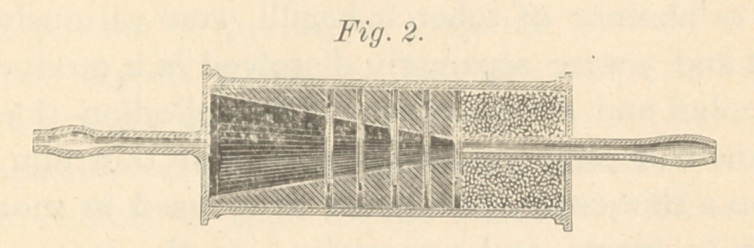# College of Physicians of Philadelphia

**Published:** 1885-04

**Authors:** W. H. Webb


### FACTS SERVING TO PROVE THE CONTAGIOUSNESS OF TUBERCULOSIS; WITH RESULTS OF EXPERIMENTS WITH GERM TRAPS USED IN DETECTING TUBERCLE-BACILLI IN THE AIR OF PLACES OF PUBLIC RESORT, AND A DESCRIPTION OF THE APPARATUS. *By* W. H. WEBB, M. D.

[Read before the College of Physicians of Philadelphia, February 4th, 1885.]

It is in accord with the spirit of the age to attempt to get at the root of all things affecting the health of our race. The causes of tuberculosis and influences modifying the progress of the malady claims not only the active interest of those engaged in the field of scientific medical investigation, but the attention of every mind that fully appreciates the universal prevalence and immense mortality resulting from the scourge consumption. It may add somewhat to the interest, if not to the elucidation, of this subject to trace the progress of thought springing from the observation of clinicians of all ages. By collecting the expressions of the *master-workmen* of different times, we will see that knowledge upon the subject has been steadily progressive, and that what now seem to be proven facts, have been preceded by flashes of truth in almost every epoch of medical history. The discovery of the tubercle-bacillus was not drawn from the inspiration of genius, but from the shaping of clinical facts gathered in the progress of our art, from the time of the Father of Medicine until the day that Koch discovered a peculiar microbe in tuberculous patients.

The word tubercle was used by Hippocrates,[1] but he applied it principally to designate small external tumors—phymatæ—as hordeolum, furunculus, sycosis, anthrax, wheals, etc. It is said that the word was used by Celsus, about A. D. 20, but no special meaning was attached to it by him.

Franciscus Deleboe Sylvius, who lived between 1614 and 1672, was not only a strong advocate of the doctrines of Hippocrates, but also did much to advance the knowledge of this disease. He was the first to use the word tubercle in designating the hard nodules found in the lungs of the phthisical. He was also the first to speak of the formation of cavities and destruction of the lung tissue by the softening and breaking down of these hard masses. He describes three kinds of consumption—one of the blood; one of the lungs, occasioned by bad nutrition; and one of degenerated glands. Through this latter proposition he may be regarded as the originator of the theory of a relationship between consumption and scrophulosis. "He believed that in the predisposed the disease my be excited by contagion."[2]

1 "The Genuine Works of Hippocrates." Published by the Sydenham Society. London, 1849.

2 Quoted in "A Practical and Historical Treatise of Consumptive Diseases." By Thos. Young, M. D. 1815. p. 178.

As a result of autopsies made by Willis, and also by Bonetus, about from 1640 to 1670, the subject of phthisis was very much advanced.

When Richard Morton's[1] celebrated work appeared, the opinions set forth therein were not only greatly in advance of those of his own time, but were destined to supplant all others, and to be accepted as correct for more than one hundred years after his death. He asserted that all consumption originated through tubercles, and that they gave rise to the dry cough. The idea entertained by Hippocrates, that consumption was due to inflammation and ulceration, he strongly opposed. He also declared that he believed the disease to be propagated by infection. "For," said he, "this distemper—as I have observed by frequent experience—like a contagious fever, does infect those that lie with the sick person with a certain taint." (p. 67.)

Desault[2] also insisted that tubercles were the essence of consumption, and that ulceration of the lungs and hæmoptysis were the result of the deposit. He believed in the contagiousness of the disease when ulceration had occurred. A statement, which at this day seems to have been almost prophetic, occurs in his writing upon Tuberculosis: "Worms," he declares, occasioned by the putrefying lungs, "propagate the disease and cause it to spread."

From the time of Morton to 1793, the subject of tuberculosis commanded the attention of some of the most distinguished men in medicine. (Sydenham F. Hoffman, Boerhaave, Van Swieten, Sauvages, Morgagni, Cullin, Hufeland, Portal, Stark, Ruysch, Stahl, Reid and Baumé.) At this time Baillie's

---

1 "Phthisiologia, or a Treatise of Consumption." 2d Ed. London, 1694.

2 Quoted by Thomas Young, M. D. Loc. cit.

great work appeared,[1] in which he demonstrated the existence of tubercles in other organs besides the lungs.

Bayle,[2] an independent worker as well as thinker, insisted most strenuously that phthisis is a general chronic disease, and owes its origin to a special principle—the tuberculous. He, too, denied most positively the teachings of Hippocrates, that consumption was due to inflammation and ulceration. To him is due the credit of discovering what is now known as miliary tuberculosis. "Out of nine hundred autopsies performed by him, he found 624 had tubercular phthisis, 185 the granular or miliary tubercle, 72 melanotic, 14 ulcerous, 4 the calculous, and 3 the cancerous." He says further: "This disease appears always to depend on a peculiarity of constitution. Hæmoptysis is a frequent symptom of consumption, and is sometimes mistaken for its cause; but it often happens that when hæmoptysis has been fatal, the lungs are found full of tubercles."

Laennec, who followed Bayle, declared that all consumptions, including scrophulosis, were nothing but the consequences of the tuberculous specific principle, which might be inherited or be acquired. In demonstrating his theory he made use of auscultation, which it is said he originated, in accurately determining the diseased condition of the lungs. Laennec's views in regard to the pathology of consumption, notwithstanding he had very strong opponents among his colleagues, were held for a long time. Schönlein, in the main, held with Laennec, but differed most positively with him in making a marked distinction between tuberculosis and scrophulosis. At this times scores of eminent investigators were busy with this sub-

1 "Morbid Anatomy." London, 1793.

2 "Recherches sur la Phthisis." Paris, 1810, p. 66. Quoted by Dr. Young. Loc. cit., p. 452.

ject, and the confusion of ideas that then existed occasioned the promulgation of many widely divergent theories; thus were described tuberculization of pus, tuberculous pus, gray tubercle, yellow tubercle, gray infiltration, tubercle granules, tubercle corpuscles, granulosis, albuminous tubercle, etc., etc. Indeed, the number of forms assumed by this disease was limited only by the number of writers upon it. This tended to give to the simplicity of the theories advanced by Laennec a great attraction to many, who held to them for sheer comfort of mind. Out of this dire confusion a way was opened by Virchow, whose cellular pathology gave us a positive science by which the theories of previous writers were exploded.

In the early part of 1882 Dr. Robert Koch made his name immortal by giving to the world the result of the researches and experiments[1] by which he swept away all false ideas that existed in regard to tuberculosis for a period of over two thousand years. It was then that he made the announcement that "tuberculosis is a specific, infectious disease, caused by a specific micro-organism—the bacillus tuberculosis—which constitutes, in fact, the true tubercle virus." This statement is one of the most remarkable, in its import, in the history of medicine.

Koch reached this position by the results obtained from experiments made with the tubercle-bacilli which he had artificially cultivated. He prepared a nutritive substance and introduced into it a speck of pus taken from a tuberculous human lung. In this way he obtained a number of bacilli, with which he infected fresh material, and by frequent repetition of this process, which he carried on for many months, he succeeded in obtaining bacilli very many generations removed from those taken from the diseased lung. These cultivated bacilli were

1 Die Etiologie der Tuberculose. Berliner Klin. Wochenschrift, 1882, No. 15.

introduced into the circulation of healthy animals, and in every instance induced tuberculosis. Tubercles in large numbers were found in the lungs, liver and spleen of all the animals thus experimented upon.

The labors of the illustrious Pasteur, of France, and of Koch, of Germany, are now well known to us all. They are the leaders of a host of equally zealous investigators who have acquired more or less distinction through their efforts in this direction.

Villemin,[1] Buhl,[2] Bollinger,[3] Fraenztel,[4] Balmer,[5] Ruhl, of Bonn,[6] Lichtheim,[7] the late Professor Cohnheim,[8] Gaffky,[9] Ewald,[10] Ehrlich,[11] Kowalski,[12] Wilson Fox,[13] Cheyne,[14] Shakespeare,[15] Sternberg,[16] Ernst,[17] Colin,[18] Tappeiner,[19] Williams[20] and others, who form a legion of self-sacrificing, earnest and conscientious workers, banded together in the interests of science and of their fellow-men, and inspired by the hope of being able

1 Gazette Med. de Paris, Dec., 1865. Also "Etudes La Tuberculose." Paris, 1868.

2 Lungenentzundung, Tuberculose und Schwindsucht. 1873.

3 Archiv. f. Experim. Pathologie, Bd. i. 1873. Also N. Y. Medical Record, March, 1884.

4 Berliner Klin. Wochenschrift, 1882, No. 45. 5 Ibid.

6 Medical Record, New York, May, 1883. 7 Ibid.

8 "Consumption as a Contagious Disease," London, 1880. Translation by H. D. Cullimore.

9 Report of the Imperial Health Office, Berlin, 1884. See Review of Amer. Jour. of the Med. Sci., July, 1884.

10 Med. News, Phila., Sept. 6, 1884, p. 275.

11 Deutsche Med. Wochenschrift, No. 19, 1882.

12 Wiener Medizinische Presse, Feb. 24, 1883.

13 Med. Times and Gazette, London, 1883, Vol. II, page 672.

14 Practitioner, London, 1883, Vol. XXX.

15 Proceedings of the Phila. Co. Med. Society, 1884, pp. 315, 320.

16 Medical Record, New York, Oct., 1884.

17 Amer. Jour. of the Med. Sci., Oct., 1883.

18 Med. Centralblatt, 1873, No. 30.

19 Virchow's Archiv, Bd. 82, 1880.

20 The Lancet, London, Feb. 24 and July 28, 1883.

at some future day to stay the progress of a malady which has been the occasion of more deaths than all the epidemics of disease, and all the disasters by land and sea, not only command the attention and support of the scientific world, but also the gratitude of every intelligent human being.

With a rapid but steady pace those observers are advancing on the road which will soon lead to the desired goal. The clouds of error are being dissipated by newly discovered truths, and to-day the subject of tubercular phthisis is better understood than ever before. It is my purpose this evening to bring to the notice of the Fellows of the College some facts by which, I think, the contagiousness of tuberculosis is clearly demonstrated.

Careful researches by De Quatrefages,[1] Cook,[2] Livingston,[3] Rush,[4] Budd,[5] and others, seem to prove that tuberculosis first appeared among the inhabitants of Europe, and gradually manifested itself in those parts of the world with which they had intercourse. If this is true, it is one of the best evidences of the contagiousness of phthisis.

A contagious or infectious disease can have but one cause, and this is eminently true of tuberculosis, which does not arise from a variety of causes, but is solely due to the tubercle-bacillus. Wherever this bacillus finds its proper nidus it will there develop and multiply; and, if this should be in living animals or human beings, the progress of the disease will be determined by the character and amount of food offered for the growth of this germ; thus with a nidus rich and plentiful we may have a case of acute phthisis lasting not more than

---

1 The Human Species, by A. De Quatrefages. N. Y., 1883, pp. 428, 430.

2 Ibid. 3 Ibid.

4 Medical Inquiries and Observations. Phila., 1789; p. 137.

5 The Lancet. London, 1867. Vol. II, pp. 451, 452.

thirteen days;[1] and, on the other hand, if the pabulum is poor and scant, the case may be a chronic one extending over a period of twenty-five years, such a case having occurred in my own practice.

The bacilli may enter the system through the lungs or by the stomach. The air we breathe, as well as the food we take, especially in the vicinity of the phthisical, may be laden with these germs. The air of the ventilating flues at the Brompton Hospital, when carefully examined, was found to contain tubercle bacilli in fair abundance.[2] The sputa of tuberculous patients drying upon our streets is ground into an impalpable powder by the feet of pedestrians, and is then disseminated through the air to be inhaled alike by the healthy as well as those predisposed to tuberculosis. Such sputa, mixed with the dirt of the street, have been collected, dried and powdered again, at frequent intervals during a period of several months. Guinea-pigs were then inoculated with this matter and in a short time the animals thus treated died of tuberculosis.[3]

To admit that the tubercle-bacillus is a pathological product is to express a belief in spontaneous generation,[4] and I feel sure that none of my enlightened hearers are prepared to subscribe to that doctrine.

It is asserted, by some pathologists, that other matter or irritant than the tubercle-bacillus is capable of producing the disease. This idea is not a new one, for Richard Morton says: "Chalky stones that are preternaturally bred in the lungs, or nails, or other hard bodies, slipping down into the

1 Medical Diagnosis, by J. M. Da Costa, M.D., LL.D. 6th Ed. Phila., 1884; p. 320.

2 The Lancet. London, July 28, 1883.

3 Med. and Surg. Reporter. Phila., 1884. Vol. I, p. 697.

4 Floating-matter of the Air. By John Tyndall, M. D. New York, 1882; pp. 277, 320.

lungs, when persons laugh, are to be recorded among the causes of a consumption of the lungs,"[1] and he narrates a case, p. 247.

It is also claimed by a number of writers that certain callings or occupations may be a cause of tuberculosis, owing to fine particles of dust inhaled by those employed. Thus coal miners, dry grinders, stone cutters, moulders, operatives in cotton mills, etc., are apt to have the disease. But those who believed that the dust breathed by individuals engaged in these occupations might occasion phthisis were evidently oblivious of the fact that the air carried, in the form of germs, far more potent factors; and while the dust may have produced an irritation of the air passages, the presence of the tubercle-bacilli was essential to the production of the disease. The inhalation of irritants, or lowered vitality, occasioned by certain occupations, may cause the predisposition, but they are never the cause of the disease *per se*.

Not all the predisposing causes united could in any instance induce tuberculosis without the advent of the tubercle-bacillus. That something more was needed was admitted by Pollock twenty years ago, when he declared that there must be "some subtle agent to precipitate, concentrate, and shape these elements of the disease into tubercle."[2] And Da Costa says, "Whatever it be, is something special.[3]"

Experiments have demonstrated, beyond doubt, that it is impossible to induce true tuberculosis in any case where proper precautions have been taken to remove from the irritant used all living germs. This is now accepted as a fact by many of those who once held a contrary opinion. Wilson Fox, Cheyne, Sternberg and others, who performed these experi-

---

3 Loc. cit., p. 67.

1 The elements of Prognosis in Consumption. London, 1865, p 337.

3 Phila. Med. Times, June 19, 1880.

ments under the conditions mentioned, have acknowledged that under such circumstances it was impossible to produce the disease.

Objections are also made to the fact that these bacilli are the cause of tubercle, because they were not found in all the cases of tuberculosis examined by certain investigators. It is fair to presume that in these instances they must have escaped detection, since bacilli have been found in every case of tuberculosis examined by careful observers.

Many instances are recorded where foreign bodies have been carried into the lungs by gunshot wounds or otherwise, without occasioning much disturbance in the parts or seriously affecting the health.

Rush,[1] with his experience of the Revolutionary War, declared that he had never known a case of phthisis to result from wounds in the lungs, and this observation was supported by the Surgeon General of the Royal Army.

A number of cases of gunshot wounds of the lungs occurred during the late war, but, as far as known, they were not the occasion of any death by phthisis.[2]

I am free to admit that, in cases where a predisposition exists, it may be still further developed by the presence of an irritant, just as a furuncle in one individual may be harmless, and in another the starting point of a cancer. The late General Baxter,[3] of this city, received a wound in the lungs on the 6th of May, 1864, and was more or less actively engaged in his

---

[NOTE. That the excretion of these bacilli might prove to be the *materies morbi*, was suggested by me some time ago; and this opinion is also entertained by Dr. G. M. Sternberg, U. S. A., who subsequently made the same suggestion in the *Med. Record*, N. Y., Oct. 25th, 1884.]

1 Medical Inquiries and Observations. Phila., 1805, Vol. 11, pp. 72, 73.

2 Med. and Surg. Hist of the War. Second issue, 1875, Part 1, Surg. Vol., p. 478, 481.

3 The Daily Evening Telegraph, Philadelphia, May 10th, 1881.

duties until twelve years afterwards, when, during a fit of coughing, he ejected what appeared to be a hardened bit of pus. This, upon examination, proved to be the envelope of a small piece of coarse, red cloth, half an inch in diameter (such as is used for the stiffening and padding of coats), which had been carried into his lungs at the time he received the wound in 1864. During all this interval there had been a constant suppuration of the lungs, occasioning considerable discomfort, but not sufficient to render him unable to fill several important positions demanding his careful attention. Three years after expelling the foreign body (seventeen years from the time he received the wound) he died, it is said, from phthisis. In this instance, admitting that he died from phthisis, a predisposition to the disease was evidently established by a greatly lowered vitality, occasioned by the long continued suppuration. For twelve years he lived without a sign of phthisis; but after he had rid his lungs of the original irritant, the bacillus tuberculosis found its way to the rich soil so long prepared for its reception, and there multiplied until the life of the individual was ended.

So certain diseases, occasioning an irritation or a lowered vitality of the pulmonary mucous membrane, have the reputation of being the indirect causes of tuberculosis. Measles, especially when occurring in children of phthisical parents, are liable to have consumption as a sequel. The mucous membranes are implicated in this disease, probably more so than in any of the eruptive fevers; the epithelium is cast off, and the denuded membrane exposed to the direct contact of the tubercle-bacillus.

It has been satisfactorily demonstrated that tuberculosis may be caused by inoculation in the human subject. Laennec, whilst examining a vertebra containing tubercle, slightly wounded one of his fingers with the saw blade. In the site of this wound a small, round tumor subsequently appeared, which, upon investigation, exhibited all the physical characters of tubercle. It was destroyed by the application of an escharotic, and no bad effects resulted from it.[1]

Another instance was that of a fisherman free from tuberculosis, but suffering from gangrene of the toe, who was purposely inoculated. Thirty-seven days after the experiment he died, and the autopsy revealed a tuberculous deposit in the lungs and liver.[2]

But the most satisfactory evidence of the effects of inoculation of tubercle is that presented in the case recorded by Dr. E. A. Tscherning.[3]

The subject, employed as cook in the family of Professor H., was a perfectly healthy woman, about twenty-four years of age, with a history unexceptionally free from any hereditary taint of scrofulous or tuberculous affections. After a short illness the Professor died of phthisis. The cook unfortunately broke the glass sputum-cup used by her employer, and a spicula from it punctured one of her fingers. Fourteen days afterward there appeared at this point what seemed to be a felon. This was treated at Professor Studgaard's clinic, and at the end of a few weeks the finger was much better; a little nodule, however, about half as large as a pea, was found to exist in the subcutaneous connective tissue, which after awhile became tender and œdematous. This was now cut out, and the wound healed readily. About three months from the time of the accident, Professor Studgaard found that the sheath of the

---

1 Diseases of the Chest. Translated by J. Forbes, M. D., London, 1834, p. 305.

2 Gazette, Med., 1872, p. 192. Quoted in Biennial Retrospect of Med. and Surg., 1871-72, p. 38.

3 Hospitals-Tidende, Copenhagen, Dec. 17th, 1884.

tendon was thickened, and two cubital and two axillary glands were enlarged. He disarticulated the middle finger, and the tendon, with its thickened sheath, as well as the enlarged glands at elbow and axilla, were dissected away. Upon examination, the sheath of the tendon was found to be filled with pale granulations. Sections of the sheath and of the extirpated glands were subsequently subjected to microscopic investigation, and numerous tubercle-bacilli found in them, which positively established the peculiar character of the affection.

Dr. Tscherning has observed upwards of thirty cases of localized tuberculosis, and in each instance the microscopical appearances were the same as in this case.

Many eminent men, by their constant attendance upon the phthisical and by their close and frequent study of the post-mortem conditions of their cases, have been made victims to the disease themselves. Among those who have met death in this way may be mentioned Bayle, Laennec, Delaburg, Dance, Young-Thomas, and many other names could be added.

Much stress has been laid upon heredity as being one of the chief causes of the vast mortality from this disease. It is my belief that phthisis is never transmitted from parent to child; it is simply a predisposition that is inherited. By predisposition I mean a greatly lessened power of resistance in the tissues, especially in the lymphatic system. If tuberculosis was inherited we might expect to find some indications of this in the fœtus, but the observations of Guizot,[1] Gluge,[2] Heller,[3] and Virchow,[4] have shown that this is not the case. In 1,300 fœtuses examined by Heller there was no evidence of a tuberculous taint in any one of them, notwithstanding

---

1 Quoted by Dr. Durant. Trans. of the N. Y. State Med. Soc., 1878, p. 174.

2 Ibid. 3 Medical News, Phila., 1884, p. 302. 4 Ibid.

the fact that in one instance the patient died of phthisis with the fœtus in utero. Virchow, with his experience of more than fifty years, says: "He had not seen a single case of direct transfer in the fœtus." This also holds true in regard to the offspring of animals which have been under observation while suffering from tuberculosis; there is no instance on record in which they have exhibited a trace of the disease.[1]

The presence of the disease in infants is undoubtedly due to the bacillus, or its spores, contained in the milk of the phthisical mother, or in the air it is constantly obliged to breath.[2] In other words, the disease is not transmitted, but it is acquired. I have elsewhere shown the fallacy of the hereditary transmission of the disease.[3]

There are but few insurance companies that will accept as a risk any one whose family history is not clear of tuberculosis; hence it would seem that such careful exclusion would remove all questions of hereditary transmission in those losses which they may sustain by deaths from phthisis. One of the most conscientious companies in this respect is The Mutual Life Insurance Company, of New York. It is especially careful in excluding such risks, and will not only refuse to accept an applicant who has a phthisical history, howsoever remote, but will not regard an application in which there is the least evidence of a predisposition to the disease, no matter what the age of applicant may be. Notwithstanding the exercise of an unusual amount of vigilance, they are nevertheless obliged to declare that "Consumption has been the occasion of more deaths than any other disease, giving a percentage of $17\frac{16}{100}$ of the total mortality; while deaths recorded under other

1 Practitioner, London, Vol. XXX, 1883, p. 318.

2 British Med. Jour., 1879, Vol. XX, p. 616.

3 "Reasons for Believing in the Contagiousness of Phthisis." Read before the Philadelphia County Med. Society, June 11, 1884.

headings, but properly belonging to this, would swell the number to 20 per cent."[4] Here, then, is a freedom from an hereditary taint as far as rigid examinations are capable of determining it. Under such circumstances the rate of mortality is surprising, to those, at least, who have faith in the hereditary transmission of the disease. A few years previous to that in which this report was made, the death rate from consumption in the adult male population of New York City was $30\frac{17}{100}$ per cent. This but little over 13 per cent. of the deaths among those who were considered especially exempt from the disease.

It would be impossible to enumerate the so-called causes of tuberculosis. The disease has been attributed to every imaginable influence which could occasion a morbid condition of the system. This error is readily accounted for when it is understood that any influence which will bring about a lowered vitality of the body will induce a predisposition to the disease, which is established by the presence of the bacillus tuberculosis.

*Age.*—In looking over "health reports" and other statistics, I have been surprised at finding records relating to the time of life when tuberculosis is most prevalent, which are entirely at variance with the ideas entertained by many practitioners. It is very generally believed that at the age of puberty, especially in those supposed to possess hereditary taint, phthisis is most apt to manifest itself, and that the liability to contract the disease is lessened with advancing years. It seems, however, that this is not the case, for in early childhood and at puberty the mortality is less than at any other period of life. There is reason for believing that the best way to determine the time of life at which the disease is the most fatal is to

4 Preliminary Report of the Mortality Experience of The Mutual Life Insurance Company, of New York. New York, 1875, p. 12.

compare the death rate occasioned by it at certain periods of life with the number of living persons at the same age. This has been done by several reliable persons, including Dr. Edgar Holden,[1] and I will read to you an interesting table prepared by A. Wuerzberg, the librarian of the Imperial Health Office at Berlin, Prussia.[2] This table is as follows:

| | | | | | | |
|---|---|---|---|---|---|---|
| Of 10,00 individuals | aged | 0– 1 year | there die annually | of consumption | | $25\frac{45}{100}$ |
| " | " | " 1– 2 | " | " | | $20\frac{41}{100}$ |
| " | " | " 5–10 | " | " | | $4\frac{66}{100}$ |
| " | " | " 15–20 | " | " | | $18\frac{37}{100}$ |
| " | " | " 20–25 | " | " | | $30\frac{24}{100}$ |
| " | " | " 25–30 | " | " | | $36\frac{78}{100}$ |
| " | " | " 30–40 | " | " | | $41\frac{12}{100}$ |
| " | " | " 50–60 | " | " | | $67\frac{94}{100}$ |
| " | " | " 60–70 | " | " | | $93\frac{18}{100}$ |
| " | " | " 70–80 | " | " | | $61\frac{72}{100}$ |
| " | " | " over 80 | " | " | | $25\frac{80}{100}$ |

This table goes to show that at the two extremes of life, where vitality is at the lowest, thus lessening the power of resistance, the disease is most fatal.

It must not be forgotten that among the many causes which lead to a predisposition to tuberculosis, conditions of mental depression play an active part. Bad habits or immoral conduct, which lead to bitter regrets; or domestic infielicity, occasioning long continued fret and worry, will produce a depression of this character more or less marked. A case recently came under my notice in which the patient's health was first affected by the unfortunate conditions of his domestic affairs; they were the occasion of continued anxiety and worriment for several years, and finally brought him into a condition of nervous exhaustion. This was soon followed by the signs of phthisis, and he died about six months afterwards. In this case the predisposition was certainly due to a lowered vitality,

1 The Medical Record, New York, July 12, 1884.

2 American Journal of the Medical Science, July, 1884, p. 192.

induced by long-continued mental depression, aided somewhat by the patient's occupation, which was that of a book-keeper. There was no hereditary tendency to the disease, and had he been more fortunate in his domestic relations he might still be living.

The following interesting cases, which I have personally investigated, will go to support some of the assertions I have made in my paper:

*Case 1.*—J. E., an invalid, was married; twelve months afterwards his wife gave birth to a child, and in the following month the father died of phthisis. At the age of five months the child died of marasmus, and in sixteen months after her accouchment the wife died of phthisis. She was of a healthy and long-lived family, but had occupied the same room with her husband during his illness.

*Case 2.*—S. Y. was a healthy young man who married a lady that was physically below par. About a year after marriage she gave birth to a child, and from this period onward she declined in health and ultimately died of phthisis five years afterwards. Eighteen months prior to her death her husband exhibited symptoms of tubercular laryngitis, and died of consumption four weeks before his wife. In this case I ascertained that the wife had come from a tuberculous family (her parents and five sisters having died from the disease), while in the husband's family there was not a trace of tuberculosis, his parents living far beyond the allotted three score and ten, and his brothers and sisters in the full enjoyment of health. This gentleman, who was greatly devoted to his wife, and had been constant in his attendance upon her and had slept in the same room.

*Case 3.*—I. R., a young man, aged twenty-seven, of very temperate and regular habits, who presented no family history of tuberculosis, and whose constitution and general health were excellent, married a young lady of delicate health, in whose family consumption had caused the death of father, mother and three sisters. The occupation of the young man was that of ticket agent in a railroad office. About three and a half years after his marriage he became ill, and a year after the commencement of his illness died of phthisis. Two years and a half susequent to his death his wife died of the same disease.

In the first case narrated to you the healthy, robust bride certainly contracted the disease from her husband. In the second case the young man, with an excellent family history and good health at the time of his mrrriage, not only contracted the disease from his phthisical wife, but died of it four weeks before she did. In the third case we have a healthy, and vigorous young man, with an excellent family history marrying a phthisical girl from a phthisical family, and what is the result? Through his close companionship with his consumptive wife he contracts the disease which occasions his death two and a half years before his wife succumbs to the malady.

I might add to this list a number of similar cases, were it necessary to do so, for I have the notes of many which prove conclusively the contagiousness of phthisis. Indeed, any one can lay his hands upon recorded cases without number which would convert even the most skeptical to this belief.

No one, not even the non-contagionists, can declare that the cases I have narrated are simply "coincidences." The experience of medical men, especially of those who are engaged in the treatment of lung disorders, must be similar to my own, and I cannot see how there can be a question in their minds in regard to the contagiousness of phthisis.

If the most convincing proof of the truth of a comprehensive theory lies in its power of absorbing and finding a place for new facts, and its capability of interpreting phenomena which had previously been looked upon as unaccountable anomalies,[1] then I know of no theory more truthful than the one which I have advocated before you this evening. It will fully explain every phenomenon connected with this malady, the universal mortality it occasions in every part of the world, and why one member of a family after another, with no hereditary predisposition, has succumbed to its power.

It is a singular fact that in all the recorded cases where the disease has been occasioned by close association with the phthisical, as in nursing, it has been unusually rapid in its course, frequently carrying off those unfortunates during the lifetime of those from whom the disease was contracted.

And what does all this teach us? Simply this, that our real strength in battling with this terrible disorder lies not so much in medication as in the application of hygienic and sanitary laws.

Surgeon General Von Lauer, of the Royal Prussian War Department, in a letter dated October 16th, 1884, kindly enclosed to me a copy of the instructions which he issued in regard to diseases of the lungs. They are of such importance that I quote them in full. It is also of interest to know that in Austria, where the bacillary origin of tuberculosis met with greater opposition than anywhere else in Europe, the Government has recognized its infectious nature and has issued official instructions similar to these of Surgeon General Von Lauer. The same precautionary measures should be adopted in the hospitals of our own country, and it is fair to assume that this will be done.

---

1 Contributions to the Theory of Natural Selection. By A. R. Wallace, London, 1870, p. 45.

[COPY.]

DEPARTMENT OF WAR.

BERLIN, Aug. 31st, 1882.

The various detailed reports which, in pursuance with request of Nov. 24th, 1881 (No. 157, II, M. M. A.), have reached this Department, have clearly shown that there exists no material difference of opinion regarding the reasons for the high annual sick and mortality rate from consumption during the time of active service.

The universally acknowledged causative relations will necessarily lead to still greater caution in the treatment and care of those exhibiting the earlier symptoms of chronic pulmonary disease, as well as those in whom a predisposition is suspected or clearly discernible. The prescribed regulations should therefore be borne in mind, in order that the number of consumptives in the army may thereby be diminished. The following instructions must always be carefully observed:—

1. Although the predisposition to affections of the lungs cannot be objectively determined, and the time permitted the surgeon during the recruiting service often not extended enough to permit a careful and searching examination, to determine this question, the medical officer in charge is earnestly urged to consider the build, configuration and exhaustibility of the thorax. In this connection he is to adhere closely to the instructions of April 8th, 1877, regarding the normal limitations. Shoemakers and tailors (Oekonomiehandwerkern) of delicate frame require very careful inspection of the chest organs.

When the circumstances attendant upon the recruiting service are not favorable to exact examination, special attention is to be paid to more rigid inquiry when the recruit reaches his regiment, as directed in Par. 13 of the instructions. Here it will be of value, in forming an opinion in your cases, to seek direct officinal information regarding family history or previous disease of the lungs or pleura.

But in order not to lose sight of those cases which have either been overlooked at the first inspection, or whose character could not then be ascertained, the recommendation of the various corps surgeons, that, with the coöperation of the proper authorities, medical examinations should be repeatedly made at stated intervals, should be particularly borne in mind, with special attention directed to those in whom disease of the respiratory organs is suspected. Special records, carefully noting the condition at each examination, must be kept. The extent to which the weakly are to be spared the arduous work of the training must be determined by the requirements of individual cases. The industrious use of douche baths, to harden the skin and accustom to exposure, naturally suggests itself here.

2. The attention of surgeons is directed to the fact that the instructions (Par. 5, Sec. 4, to Par. 7, Sec. 2) do not permit a judgment upon volunteers without considering their fitness for field service. Complete fitness, therefore, is indispensable to a declaration of efficiency.

3. For convalescents from acute disease of the respiratory organs a prolonged period of after treatment and care is desirable. If the circumstances of the patient make home attention attainable, and only then, is a lengthy furlough to be recommended. Those returning from such furloughs are to be carefully reëxamined, and, if necessary, their transference to the appropriate health resort taken under advisement.

4. That the first symptoms of disease of the lungs may not be overlooked in making the round of the barracks, particular attention should be paid to apparently mild "catarrhs," utilizing, if necessary, evening temperature measurements. Doubtful cases should be transferred to hospital for observation.

5. The opinion of many medical officers, that prompt measures should be taken for the discharge of sufferers from chronic pulmonary disease, should not be forgotten. That even one attack of hæmorrhage (Bluthusten), if it is proven to be of undoubted pulmonary origin, is sufficient cause for discharge, and is especially emphasized. That the early dismissal of cases affording no probability of usefulness to the service removes a source of infection for hospital and barrack, must be viewed as by no means the least important advantage of this provision,

Now that experimental pathology has furnished exact scientific corroboration of the theory of infectiousness of phthisis, more importance than ever must be attached to the separation, both in hospital and barrack, of those afflicted with or suspected of phthisis, from other patients, especially from those suffering with inflammation of the lungs or recent bronchial catarrh. The sputum being the principal carrier of the disease germ, and consequently the principal source of infection, provision for its removal and disinfection (Unschadlichmachung) follows as a matter of course.

In answer to the question raised by this Department, as to whether new measures for the diminution of the number of cases of phthisis, with particular reference to the necessity for the establishment of climatic summer or winter stations for their treatment, were called for, the responses were unanimous against such establishment. The indications for them were considered uncertain, and the existing provision adequate for the present necessities of the army. The Department endorses this view, and is convinced that the careful observance of the general directions herewith transmitted will be of interest and service to the army as well as to the patient. Although tedious attempts at cure by long-continued stay at climatic stations may be considered of doubtful value to the phthisical patient, and not at all likely to furnish the army with a soldier fit for field service, the prompt despatch of a convalescent from an acute non-phthisical affection of the respiratory apparatus to an appropriate station, is warmly to be recommended. Such station, from among those at the disposal of the Department, is to be carefully selected, and treatment conscientiously carried out.

This communication, with five copies, is transmitted to you, with the request that you submit your views to the General Commanding, and instruct the sanitary officers of the corps to be guided thereby. (Signed) V. LAUER-STRUBE,

*Department of War, Army Medical Division.*

To all Royal Corps Surgeons, No. 20, 4, 82, M. M. A.; 64, 9–84, M. M. A.

Like other disease germs the tubercle-bacilli are carried by the air, and will, of course, be found to be more plentiful in the vicinity of the victims of tuberculosis. A single bacillus may as surely induce the disease as the presence of a great number; and since we are at no time free from the chance of inhaling this germ, our safety lies in avoiding a "predisposition" to lung troubles. In order to determine whether the bacilli might be readily found in the air of the street, or of places of public resort, I have constructed the instruments which I now show to you.

Fig. 1

The first apparatus I had made was after the plan of the ordinary inhaler (as shown in the accompanying engraving, Fig. 1). The long tube (E) passed into a little well (W) at the bottom of the bottle containing glycerine, which was intended to retain any germs carried by the air passing through it; by rotating the bottle its sides were also smeared with glycerine, to give a still larger surface of glycerine for the contact of the air which, after entering the funnel (B), was forced through the apparatus by using the pump (D). This was undoubtedly an effective germ trap, but the impossibility of drying the glycerine, which it was necessary to do in order to obtain microscopic proof of the presence of bacilli, obliged me to devise another method of obtaining them.

The second trap consisted of a brass cylinder containing a series of snugly fitted steel discs (as shown in the illustration, which gives a sectional view, Fig. 2). Each disc was perforated in such a manner that, when placed together, the

*Fig. 2.*

openings formed a cone. Between each of these discs, across their openings, thin layers of pyroxylin were placed; the discs were then introduced into the cylinder, which was tightly fastened. To one end of the cylinder the pump connection was affixed, and the other end was connected with the funnel, which was placed over the ventilating flue, to take the air. When the pump was put in motion it drew the air through the apparatus and necessarily through the veils of pyroxylin held in position by the metal discs, the pyroxylin thus serving to intercept the passage of any germs.

With this apparatus I visited a number of places of public resort, and through the courtesy of those in authority I was given free access to the parts of the establishments wherein the exit flues were located. These flues, in all instances except one, were placed in the ceiling of the auditorium and directly over the audience. Here I placed the funnel-shaped extremity of the apparatus, and its pump was kept continuously in motion until fifteen or twenty minutes after the audience had retired. This experiment was repeated a number of times at each establishment I visited. The trap was then dismantled; the thin layers or veils of pyroxylin were removed from between the steel discs, and placed in the hands of Drs. E. O. Shakespeare and Morris Longstreth, for microscopic examination. These skillful microscopists have made the following reports:

PHILADELPHIA, Feb. 3d, 1885.

*Dear Doctor:* The specimens of pyroxylin (Nos. 1, 2, 3 and 4) which you sent to me to examine microscopically for the presence or absence of tubercle-bacilli, were variously treated. Nos. 1, 2 and 3 were separately dissolved in a mixture of absolute alcohol and strong ether. The collodion thus formed was handled in either of two ways: *a*, a thin film was deposited on a thin cover glass, such as is used in mounting of microscopic objects, and was stained in the manner recommended by Koch for the demonstration of the tubercle-bacillus; or, *b*, the collodion was excessively diluted in a test-tube, by addition of relatively large quantities of alcohol and ether, and then allowed to stand for some hours, in order that suspended portions might fall to the bottom. The fluid was then carefully drawn off. The sediment at the bottom of the test-tube was mixed with a drop or two of sterilized beef-peptone-fluid, such as I keep in stock for bacteria-culture use, and was spread in a thin film upon a cover glass. This film was also treated in the manner above mentioned for the demonstration of the tubercle-bacilli. I had, however, considerable trouble in following these methods. There was great difficulty in decolorizing the film; many times this seemed quite impossible.

In these three specimens of pyroxylin I found no bacilli tuberculosis.

No. 4 I determined to treat in another manner. I employed two different methods: *a*. I took portions of the pyroxylin and stained it, as I would do sections of tissue in which I wished to seek for tubercle bacilli, namely, in the manner recommended and practiced by Koch. Methyl-violet being the color used in the aniline oil mixture. These were subsequently mounted in balsam in the usual way without converting them into collodion. *b.* Other portions of the pyroxylin were stained with fuchsin as the color of the aniline oil mixture. After staining in the usual way, including the methyl blue as contrast color, the pyroxylin was placed on an object-glass slide for the microscope and converted into collodion by using a mixture of ether and alcohol. As soon as it was dissolved a thin glass cover was placed over it. This latter method, in my hands, was by far the most satisfactory.

In the portion of pyroxylin prepared by the "*a*" method, I found two objects which, by their size, shape and color, had they been isolated and seen in sputum, I would have taken for tubercle-bacilli, but these objects were attached to fibers of pyroxylin which, in spite of the successive action of weak nitric acid and of strong alcohol, and in spite of the subsequent use of Bismarck brown as a contrast color, were also tinted violet. This observation must, therefore, be classed as negative, or, at least, doubtful.

In the portion of pyroxylin treated by the latter method, "*b*," I found, after painstaking search, one bacillus, which, on account of its size, shape, and quite characteristic color (bright red, the ground being blue), I had no doubt was a tubercle-bacillus. There were two other rod-like forms which in size and shape appeared identical with tubercle-bacilli, but the color which they showed was so indistinct that it could not be safely made out. I have to report, then, the finding of one tubercle bacilli in the specimen marked No. 4. There were, of course, numerous other objects in all the specimens examined, but as you wished only to know of the tubercle-bacillus, I have thought it needless to particularize concerning them. Yours very truly, E. O. SHAKESPEARE.

*To Dr. W. H. Webb.*

PHILADELPHIA, Feb. 4, 1885.

*Dear Doctor:* In compliance with your request I enclose you the following report on the examination of pyroxylin from your germ-trap, in relation to the presence or absence of the bacillus of tuberculosis (Koch).

The material consisted of five small pledgets of cotton, contained in a small phial, sealed with paraffine.

The five portions were carefully kept apart and examined separately.

The staining method employed was that recommended by Koch: Aniline oil and fuchsin, bleached with dilute nitric acid, washing with dilute alcohol, contrast stain with methyl blue (in some slides), and washing finally in absolute alcohol. The only variation made in this method of mounting as usually practiced was in using a dammar medium instead of Canada balsam, which I have employed, since I have found that the dammar hardens more rapidly than the other. The examination of the specimens can be made with the oil immersions lenses more promptly, without the risk involved in displacing the cover glasses, should the oil come in contact with the mounting medium. It was found by a preliminary examination that four of the five specimens of cotton were not likely to furnish any number of bacilli, and the further search among these four specimens was consequently abandoned.

The fifth specimen, labeled No. 1, engaged the sole attention of further examination, as it was composed of the cotton which first met the current of air as drawn through the trap.

The cotton was very much discoloured by dust and other matter, particles of which could easily be shaken off from it. Care, however, was observed so as to lose as little as possible of these adhering matters.

The staining, bleaching and other steps in the mounting were carried out by first placing the cotton in a watch glass containing the aniline fuchsin stain and allowing it to remain, tightly covered, for twelve hours. Portions of the cotton were then thinly spread on a cover-glass, and the subsequent steps of the operation carried on in this position. It has been usual,

I believe, in examining gun-cotton, to detect the presence of objects capable of being shown by a differential staining, to convert the cotton into collodion by admixture of ether and alcohol. This method I avoided, in the chief examinations, as being essentially faulty, since if the bacilli should be present in a dried film of collodion it would be impossible for the staining agents to come in contact with the micro-organisms buried in the depth of the film.

Very considerable difficulties and much tedious searching were encountered in the microscopic examination, owing to interlacing and overlying arrangement of the cotton fibers. For although the strongest pressure was placed on the covers which the glass would stand—and many specimens were lost in this manner—nevertheless the depth of the material presented a field of much confusion. The confusion was somewhat lessened, but not removed, by adding another step to the process of mounting, viz., by treating the cotton after staining and bleaching with a mixture of ether and alcohol, for the purpose of converting it into collodion. While this treatment dissolved the cotton fibers still some fibers of flax and wool were left. It did not, of course, help the confusion due to large amounts of dirt particles which were present. It was hoped that by thus making a collodion of the gun-cotton after the staining process was completed, some advantages might be obtained. Such, however, was not the case. The examination of six slides from specimen No. 1 gave the following results:

Slide a. 1 bacillus, 1 doubtful.

" b. 6 bacilli.

" c. 3 "

" d. 1 bacillus.

" e. none.

" f. uncertain.

It is not intended to convey the idea that these were the only bacilli present. A very careful examination might reveal the presence of more organisms. For the uncertainty of the examination excuse must be found in the nature of the materials dealt with; the impossibility of rendering the layer of material of uniform thickness, as can be readily done with sputa and with sections of tissue; the very large amount of dust particles scattered through a layer of considerable thickness; the facts, also, which I have not seen alluded to previously, that many fibers of cotton have in them clefts, which retain staining material in spite of bleaching; many of these clefts closely approach in length and breadth the figures of the bacillus; and, finally, the short time which I have been allowed for the work since the specimens were placed in my charge for examination.

Yours very truly,

MORRIS LONGSTRETH.

*To Dr. W. H. Webb.*

The layer or veil of pyroxylin through which the air from the flues first passed seems to have stopped the passage of all germs and other atoms, and in this way acted as a trap, to the exclusion of the other veils of pyroxylin placed between the discs for that purpose. Unfortunately, the portion submitted to Dr. Shakespeare for examination was not of the first layer, and to this may be attributed his inability to find more than one bacillus.

Furthermore, the number of bacilli found by Dr. Longstreth in the minute particle of the material he examined seems to indicate the presence of vast numbers of these germs in the entire layer removed from the trap.

And now, in conclusion, I desire it to be understood that I have spoken, not so much to maintain a proposition as to reveal the truth; and that in giving you the opinions of those who have beaten a path wherein we may the more easily travel, I have but done justice to a class of men equally endowed as ourselves to observe and to reason from cause to effect. I would also state that the aim of this paper is simply to emphasize facts, leaving you to deal with them as your wisdom may dictate. A careful analysis of the writings and investigations of those who have given special thought to the subject which I have treated, reveals the fact that since the time of Hippocrates there has been a gradual but steady progress towards the grand beacon which now illuminates our way. The very slowness of the advance, the suspicion with which the announcement of every new development has been received, and the earnest criticism to which they have been subjected, insures the safety of our position to-day. Apart from the ocular demonstration of scientific investigations of modern times, and from a purely clinical standpoint alone, the weight of evidence as to the contagiousness of tuberculosis must certainly be appreciated by you all. Even those who do not acknowledge it in words, proclaim it by their manifestation of doubt, and quiet avowal that there is something lacking which will enable them to fix upon the cause of a disease maintaining such marked characteristics from age to age, and among all people.

We are living in a scientific age, and the medical profession is thoroughly imbued with its spirit and import. We deal with facts, and are little inclined to give heed to that which is purely speculative. Such superstitions as the "Royal Touch" belong to a departed age. "Coincidences" and "Happened so's" serve no longer to answer our inquiries concerning the causes or nature of disease. Never before have we been so well established in respect to the means and methods of making research and experiments in the domain of medicine, and never before have the searchers after its truths been more earnest in their efforts or more hopeful of grand results. The discovery of the tubercle-bacillus is a scientific fact; all, with the same facilities, may see what others have seen. It is the one thing tangible, describable, known by its peculiarities among entities as readily as one individual is known from another. To doubt its existence in tuberculosis, is to doubt the utility of scientific medical research, and to abandon further progress to the unstable dreams of theorists. The sputa of the phthisical contain these germs; the air they exhale is loaded with them or their spores, and introduction into the system of animals will always produce tuberculosis *while nothing else will.* These are not speculations, but demonstrable facts! Furthermore, clinical observations go to prove conclusively that healthy individuals, living in an atmosphere contaminated by the phthisical, will contract this disease, and not any other which might be due to a lowered vitality, from being in close quarters and breathing a vitiated air. That there is yet much to be learned in regard to the tubercle-bacillus there can be no doubt. Still, having made a wide breach in the walls that hemmed in the mystery of tuberculosis, it behooves us to press on to its complete solution.

And now, Mr. President and Fellows of the College, my remarks on the question at issue are, for the present, at an end; but I feel that I would be recreant to the cause I have espoused did I not avail myself of this opportunity to state that, in more than one instance, in articles recently published, the non-contagionists, it seems to me, have willfully, unhesitatingly, and without warrant, perverted the language, even absolutely falsifying the statements, of authors they quote in support of their cause. That such reprehensible practices should be resorted to, for what must necessarily be but a momentary triumph, is of itself strong evidence of the vulnerability of their position, and requires no word of condemnation from me; nor would I think proper to notice it at this juncture, were it not to point out the necessity for all conscientious investigators to verify every and all citations by referring, wherever possible, to the original documents. And if my feeble efforts have, in the slightest degree, advanced the cause of truth and humanity, my labour has not been in vain. Now—

> "Say as you think, and speak it from
> your souls."
> "What you do
> Still betters what is done."

## Figures and Tables

**Fig. 1 f1:**
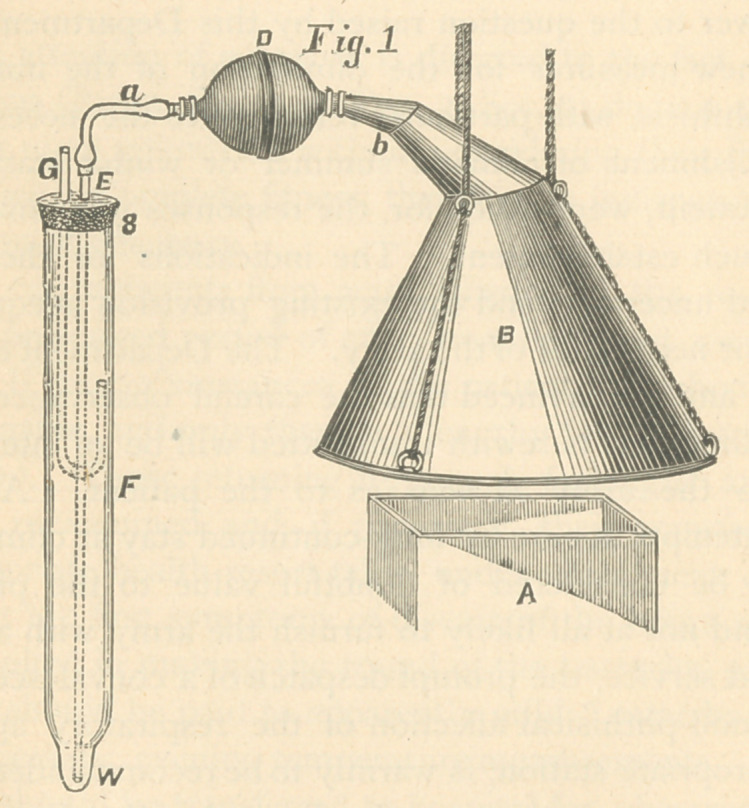


**Fig. 2. f2:**